# Previous experiences and emotional baggage as barriers to lifestyle change - a qualitative study of Norwegian Healthy Life Centre participants

**DOI:** 10.1186/s12875-015-0292-z

**Published:** 2015-06-23

**Authors:** Ingrid S Følling, Marit Solbjør, Anne-S Helvik

**Affiliations:** Department of Health Sciences, North-Trøndelag University College, Røstad, 7600 Levanger, Norway; Department of Public Health and General Practice, Faculty of Medicine, Norwegian University of Science and Technology, Post box 8905, 7491 Trondheim, Norway; Department of Social Work and Health Science, Faculty of Social Sciences and Technology Management, Norwegian University of Science and Technology, Dragvoll, Edvard Bulls veg 1, Bygg, 7491 Trondheim, Norway; St. Olavs University Hospital, Trondheim, Norway; Norwegian National Advisory Unit on Ageing and Health, Vestfold Hospital Trust, Tønsberg, Norway

**Keywords:** Primary healthcare, Health behaviour, Lifestyle change, Multi-comorbidity, Overweight, Qualitative research

## Abstract

**Background:**

Changing lifestyle is challenging and difficult. The Norwegian Directorate of Health recommends that all municipalities establish Healthy Life Centres targeted to people with lifestyle issues. Little is known about the background, experiences and reflections of participants. More information is needed about participants to shape effective lifestyle interventions with lasting effect. This study explores how participants in a lifestyle intervention programme describe previous life experiences in relation to changing lifestyle.

**Methods:**

Semi-structured qualitative in-depth interviews were performed with 23 participants (16 women and 7 men) aged 18 – 70 years. The data were analysed using systematic text condensation searching for issues describing participants’ responses, and looking for the essence, aiming to share the basis of life-world experiences as valid knowledge.

**Results:**

Participants identified two main themes: being stuck in old habits, and being burdened with emotional baggage from their previous negative experiences. Participants expressed a wish to change their lifestyles, but were unable to act in accordance with the health knowledge they possessed. Previous experiences with lifestyle change kept them from initiating attempts without professional assistance. Participants also described being burdened by an emotional baggage with problems from childhood and/or with family, work and social life issues. Respondents said that they felt that emotional baggage was an important explanation for why they were stuck in old habits and that conversely, being stuck in old habits added load to their already emotional baggage and made it heavier.

**Conclusions:**

Behavioural change can be hard to perform as psychological distress from life baggage can influence the ability to change. The study participants’ experience of being stuck in old habits and having substantial emotional baggage raises questions as to whether or not Healthy Life Centres are able to help participants who need to make a lifestyle change.

## Background

Lasting lifestyle change requires a substantial time investment. Individuals who want to take part in interventions that will enable them to change their lifestyle may encounter barriers to starting the process. Over the last few decades, there has been increasing interest in Norway in enabling people to make lifestyle changes [1–3]. In 2011, the Directorate of Health recommended that all municipalities establish Healthy Life Centres [4].

Healthy Life Centres are a service offered by the primary health care system that target people who need to change their lifestyle in terms of physical activity, diet and tobacco use to improve their health or prevent unhealthy lifestyles [4]. The background for creating Healthy Life Centres was to counteract the growth in lifestyle-related disease in the Norwegian population. The increase in individuals that are overweight, obese and have type 2 diabetes, coincident with reductions in physical activity and increased high-caloric food intake, are among the most important changes in the Norwegian population from 1986 to 2010, as measured by the population-based HUNT study [5]. In spite of these developments, there has also been an improvement in the overall health of the general population, but health behaviour varies systematically with social background [6]. Lifestyle-related diseases show a clear social gradient, where social inequalities represent a growing problem [7]. Studies that examine interventions to reduce lifestyle-related diseases have shown little effect on primary outcomes, such as for type 2 diabetes [8] and are burdened with high dropout rates [9]. The results from a Healthy Life Centre study in Norway [10] were in line with international findings [11, 12], although participants had increased their physical capacity and health-related quality of life when the intervention ended [10].

The theoretical foundation for Healthy Life Centres is a salutogenic approach that aims to help participants gain confidence in their own resistance resources and increase their ability to change their lifestyle [4]. Lifestyle change depends on individual behavioural factors. Multiple individual factors as social, psychological and practical barriers can make lifestyle change hard to perform [13]. Performing lifestyle change and adherence to change is a continuous process and contains several different phases [14]. Behavioural change theories have not in particular dealt with psychological or emotional distress [15–17], although they present several are common factors that are important for lifestyle change: social relations, attitude, stages of change and self-efficacy [18]. Individual barriers to lifestyle change may be countless and they can be hard to address by health care providers [19]. Previous negative life experiences could cause psychological or emotional distress [20] and are known to negatively affect the ability to change lifestyle [21]. Addressing psychological and emotional distress as barriers to lifestyle change may help improve outcomes from lifestyle change programs. To the best of our knowledge, the relevance of background characteristics of participants, including their life experiences, intentions and expectations, has not been previously explored. The aim of this study is to examine how participants in a lifestyle intervention programme describe previous life experiences that are important for lifestyle change when they entered Healthy Life Centre intervention programmes.

## Methods

### Design

A qualitative study was designed to explore how participants described previous life experiences that they identified as important for lifestyle change upon entry into a Healthy Life Centres intervention programme.

### Interviews and interview guide

Individual in-depth interviews were conducted from February 2013 to June 2013 at two Healthy Life Centres in Central Norway. The interviews lasted between 15 and 78 min, with a mean time of 42 min. The first author performed the interviews. The interviewer wrote down additional notes and reflections right after each interview.

A semi-structured interview guide was used to ensure that all aspects addressing the questions in the study objective were covered. The interview guide included the participants’ reasons for attending Healthy Life Centres, their previous lifestyle and their expectations upon starting an intervention programme for lifestyle change.

### Healthy Life Centres

Healthy Life Centres offer physical activity as both in- and outdoor optional activities two to four times a week, and as either individual or group-based activities with a physical therapist. The centres also offer a healthy diet course composed of five, two-hour sessions. A tobacco cessation programme is offered both as individual sessions and group-based courses that are run six times. Healthy Life Centre personnel practice principles of motivational interviewing with a focus on exploring ambivalence and helping participants to change. An intervention period lasts for three months, with the ability to extend the period two additional times for a total of nine months altogether. General practitioners can refer patients to a centre, or participants can contact a Healthy Life Centre themselves. Healthy Life Centres serve as a low threshold health service, and Norway’s public health insurance covers the cost of participation in centre programmes.

### Recruitment and participants

All Healthy Life Centre participants were eligible for the study, although there was a preference for individuals who had just begun attending an intervention programme. Personnel working at the Healthy Life Centres helped in the recruiting process. A strategic sample of informants, based on their age and gender were invited to the study at a mandatory health conversation. The personnel informed the participants that they helped recruiting and that they as personnel were not part of the study. The personnel also told that if they participated in the study or not, it would not affect the participants period at the Healthy Life Centre. Recruitment proceeded continuously until data saturation.

Altogether, twenty-three participants aged 18–70 years, with a majority of women, participated (see Table 1). More than half of participants received a referral from their general practitioner to the centre (52 %), while the rest had contacted the Healthy Life Centre on their own (48 %). Multi-comorbidities were common (91 %) among participants, with a range from zero to eight different diagnoses. Participants frequently reported lifestyle-related diagnoses such as overweight and obesity (83 %) and type 2 diabetes (22 %). These illnesses were prevalent and multiple diseases were often found concurrently in the same individual. Participants also reported muscle- and skeletal diseases (30 %) and psychological issues (26 %).

**Table 1:** Informant characteristics

Characteristics	Number of informants
Gender	
Females	16
Males	7
Age	
18–29	4
30–39	4
40–49	5
50–59	5
>60	5
Ethnicity and language spoken	
Norwegian	23
Civil status	
Single/Separated	9
Partner/Married	14
Education level	
Some college	4
No high school diploma	4
High school graduate	11
Bachelor’s degree or higher	4
Work status	
Not working^a^	13
Working < 50 %^b^	4
Studying or working > 50 %	6
Previous attempts to change lifestyle	
Specialist health care interventions	7
Primary Health care interventions^c^	8 (+7)
On their own^d^	8 (+8 + 7)
Started at the Healthy Life Centre	
Less than three months ago	15
Three months ago	7
Six months ago or more	1

^a^Three were retired at the age of 62 years or more

^b^The percentage varied from 13 % to 50 % in workload

^c^Those who had been in specialist health care [7] interventions had previous tried interventions with their general practitioner or other primary health care personnel

^d^Those who had been in specialist health care [7] interventions and those who had tried primary health care interventions [8] had earlier attempts on their own to change lifestyle

Participants had previously tried to follow advice from their general practitioner regarding diet, smoking and physical activity. In addition, participants commonly reported unsuccessfully participating in different kinds of weight loss programmes at rehabilitation centres, or with the help of community psychiatric nurses, physical therapists, and general practitioners. Four of the participants were attending the Healthy Life Centre while were waiting for approval for a gastric bypass or other specialized health service. Fifty-seven per cent received benefits or help from the Norwegian Labour and Welfare Administration.

### Ethics

All participants received both oral and written information so they could make an informed choice about participating in the study. All participants signed an informed consent form before the interview started. The Regional Committee for Medical and Health Research Ethics in Central Norway approved the study (REK nr 2012/1755).

The interviewer aimed to be friendly and non-judgmental during the interview. This is an important way of creating trust and a good relationship during the interview situation [13]. The interviewer used many interaction strategies to create friendly feelings and intimacy, especially with respect to sensitive questions and themes [22]. All participants were offered to read their transcribed interview. Only three participants accepted, and none had any comments to the transcript. Participants were also offered to know more about the content of the analysis, but none was interested

### Data analysis

Audio recordings of all 23 interviews were transcribed verbatim. MindJet MindManager 2012 was used as a systematization tool during the transcription process to identify themes. Interview notes were added to the mind map. The data were analysed using systematic text condensation with a search for issues that described the participants’ responses. The systematic text condensation were performed by looking for the essence, and thereby aiming to share the basis of the informants life-world experiences as valid knowledge [23]. The first author read all of the material, transcribed interviews and interview notes. The co-authors read three interviews, then met and discussed preliminary themes from each interview. This approach was used to assemble different opinions and perspectives, and to allow question to arise regarding differing interpretations of what the material meant [24]. The material was examined and broken down into codes, and then the codes were compared, conceptualized and categorized. NVivo 10.0 was used to sort meaning units derived from the material into codes. Material with the same content in the text was coded together. The mindJet MindManager mapping with preliminary themes that arose during transcription were added during the coding process. The authors discussed, compared and merged codes. Different conceptions of the codes were also discussed, and then the codes were reorganized until authors came to a consensus on the main themes and sub themes. The quotations in the text have been translated from Norwegian to English.

## Results

**Fig. 1:**
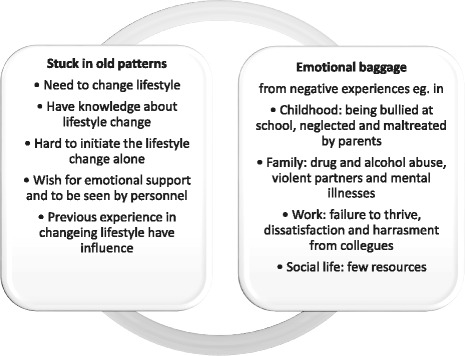
Main themes and sub-themes participants highlighted when entering a lifestyle intervention program at Healthy Life Centres

The participants’ perceptions of barriers to lifestyle changes were categorized into two main themes: they were stuck in old behaviours and habits (main theme 1) and that they were burdened by emotional baggage from previous experiences (main theme 2). Interrelated content was included in sub themes (see Fig. 1). Participants who came to a Healthy Life Centre also said they found it hard to know where to begin to change when there were so many difficult elements in their lives.

### Main theme 1: stuck in old habits

The theme “stuck in old habits” described the situation the participants faced when they began attending the intervention programme. Participants described old habits as barriers to change, including behaviours such as unhealthy food habits, being sedentary, being addicted to tobacco, and often choosing unhealthy behaviours in everyday life. They needed to change their lifestyle because their lives were adversely affected by their behaviours, which in turn affected their surrounding environment, including their children, grandchildren and work. They said they felt their lifestyle had gone in a direction that led them to ask for help to change. Participants also used the phrase *“I should have…”* several times during the interviews:

*“Why do we have these kinds of habits? I have to try to find the key to change the bad habits I have gotten into. I should lose some weight. I should quit smoking. I should work out more. Everything I do is wrong. I have to start somewhere…”* Woman *40–49 years*

A common sentiment among participants was the appreciation for being able to get help from the Healthy Life Centre. They also commonly expressed the hope that they could turn their negative health behaviours around. The personnel’s friendliness was also mentioned. Participants who had lifestyle diseases said they wanted to have a healthier life and lose weight. Those few who did not have any lifestyle diseases were concerned about gaining weight and developing cardiovascular disease and type 2 diabetes.

### Knowing, but not doing

The participants said that lack of knowledge was not the reason for failing to change their lifestyle on their own, but that the barrier was knowing how to start a change that would last. They found it paradoxical that they knew what to do, but were unable to act. They expressed hope and had the motivation to change their lifestyle, but did not initiate changes on their own. They saw the Healthy Life Centre intervention as a solution for their need for help and an opportunity to get started with activities that could work for them. One said:

*“I know a lot about good habits and things like that. I’m not clueless as to why I am so big. I know that I do not exercise, I eat unhealthy food, I know it and I know that it is not good for me. But I struggle with why I cannot do the right things when I know all this.” Woman 18–29 years*

### Previous experiences in changing lifestyle

The participants’ previous attempts to change their lifestyle became barriers to new attempts to change without supervision. At the same time, the previous attempts made them realize that at the beginning of earlier interventions they had expected the change to provide visible results quickly. Without any visible or measurable changes, they lost motivation after only a short period of trying. The participants found that there were no easy solutions to making lasting lifestyle changes. They stated their desire for long-lasting, permanent change, and said they did not want a temporary solution that would ultimately fail.

*“I have started cures and programmes for exercising and dieting numerous times, but I expected everything to happen quickly. Now I am more aware that I need to start at the beginning and take one step at time. I want this to be a permanent change….” Woman 40–49 years.*

The participants highlighted the importance of knowing that breaking from a newly changed good habit did not mean that the whole “good habit” pattern was destroyed. They asked for emotional support and to be seen by the Healthy Life Centre staff. These experiences were seen as important in giving them the courage to start and to continue gradually with healthy changes, and to be helped over time to make a long-lasting lifestyle changes. Most of them said that three months was too brief for them to succeed in permanently changing their lifestyles, given their starting point, and expressed the need to extend the intervention.

#### Main theme 2: Baggage from previous life experiences as a barrier to change

Participants explained that they were “stuck in old habits” because of previous life experiences that prevented them from initiating changes. Participants highlighted previous trauma or long-lasting bad circumstances as contributing to their need for a lifestyle change. Their emotional baggage included negative childhood experiences as well as other family and social experiences, which were accompanied by medical, psychological and social problems. One participant exemplified this complexity:

*“I have so much emotional baggage from my life that explains this bad pattern I have gotten into. I have been used as a doormat all my life. I don’t want this anymore. I had problems at my work where my colleagues harassed me, and I had problems in my private life and with bad childhood experiences. Then I lost my job. I struggled with migraines, and after all of these experiences, I was very tense. My general practitioner said that he assumed that I was depressed, and I was given medications, which caused me to gain weight, and I was somewhat overweight before. Now I have to lose weight and I can’t do this by myself, I need help…”Woman 40–49 years*

### Childhood experiences

Several participants described childhood problems as a part of the explanation as to why they were needed help with lifestyle issues. These participants commonly described difficult childhood experiences. Some mentioned bullying at school during their childhood and youth and they said that they had had a hard childhood because their peers had tormented them. Another common problem was parents who were not affectionate or who were verbally abusive or dissatisfied with them. Hard feelings and emotional distress linked to childhood memories made their adult life difficult. One participant said that ever since she started school and on into her adolescence, her father had told her how stupid she was. She said that the feeling of not being good enough had stayed with her all her life. Another individual described the difficulties caused by an overly critical parent:

*“When I hear his name I get tears in my eyes. All I have wanted my whole life is for him to be happy with me. He has always criticized me, everything I’ve ever done has been wrong…” Woman 30–39 years*

Participants had clearly not come to terms with the burdens of their life experiences and the accompanying feelings from childhood; this lack of closure influenced participants’ everyday life and burdened them, which in turn made making a lifestyle change hard to initiate and demanding to pursue.

### Family, work and social life

Participants said they found criticisms from their current-day surroundings and social life to be debilitating. They commonly described negative experiences with family, friends, fellow students and colleagues. This could take the form of harassment at work, mental problems within their family, and violent partners. Some had histories of being bullied at their present workplace. Table 2 presents the issues participants listed concerning their family and social life.

**Table 2:** Quotes on life burdens

Participant	Quotes on family, work and social issues
Female 40–49 years	“I was bullied at my work place by almost all colleagues. I was also bullied in younger years. I think it makes me more vulnerable, all those memories from earlier bullying came alive with this new bulling at my work place. I have been crying a lot…”
Female 40–49 years	“I don’t have any contact with my parents. I ended up with an abusive man. When I found myself a new man it was also a violent relationship....” In addition, one of my children have ADHD and I have been struggling a lot with all these things…”
Female 40–49 years	“My daughter is struggling in the same way as me with psychological problems and suicidal thoughts. I have several suicide attempts behind me. My daughter and I like to be at home by ourselves and I think that because of our problems we do not socialize much…”
Male 50–59 years	“When I first got married it turned out my wife was an alcoholic and her doctor said that she had little left time to live if she continued drinking like that. So with all the uncertainty with my wife being an alcoholic it all got messy. But I have to help her and be there for here, I cannot just run away…”
Male 50–59 years	“My daughter is a drug addict and I have tried to help her many times, knowing she would take all my money and run away again as she always does. Now I have not seen her in a while…”
Female > 60 years	“My daughter was abused as a child and she had many problems growing up, but it was as an adult she came forward and told me what had happened. She is mentally ill because of what happened and I have to be there for her and help her as she is often hospitalized. I have been feeling a lot of guilt and I do not understand that the abuse could have happened without me not knowing…”
Female >60 years	“I moved from my first husband because he was violent. I felt that it was better for the children to grow up with only their mother. Then I got a new husband and he had mental problems, which was demanding. I have now lost my husband and I also lost my boy, he became a drug addict …”

These experiences affected participants’ current life, and as they explained it, were a part of the reason why they were stuck in old habits. The continuing problems added to the heavy emotional baggage they already struggled with, which in turn made it hard to initiate a lifestyle change.

## Discussion

The participants who entered intervention programmes at Healthy Life Centres were stuck in old habits; making unhealthy choices with food, activity and tobacco in their everyday life, which affected themselves and their environments negatively. They wanted to improve their lifestyle but were not able to act on the knowledge they possessed about healthy habits. Previous failed experiences with lifestyle change held participants back from making new attempts on their own, and made them realize that it takes time to establish lasting changes in habits and lifestyle. In their experience, there were no easy solutions. In addition, we found that participants had significant emotional baggage from being bullied in childhood and harassed at work, not having supporting parents, having violent partners and children with drug problems. This emotional baggage was an important reason for why they were stuck in their old habits and inversely, being stuck in old habits added to their already substantial emotional burden.

### Establishing new habits in everyday life

Participants in lifestyle intervention programmes that are focused on nutrition and physical activity often go back to old habits after the intervention period [15, 16]. It appears that everyday life tends to get in the way of maintaining healthy habits [25, 26], and that it can be difficult for individuals who explicitly want a healthier lifestyle to consistently make the right choices and habits [27]. When physical activity and healthy diet became ingrained habits, participants could define themselves as successful, and the habits and their improved self image became a part of their new everyday life [28]. However, other studies have shown that many obese individuals still rely on ‘quick fix’ strategies in their struggle for permanent change [29]. The participants in our study saw the need for long-term lifestyle change, in part because several of the participants had made numerous previous attempts to lose weight. To get a referral from the general practitioner to the Healthy Life Centre, as half of the participants had gotten, may have had implication for these participants readiness for change [30]. The other half who had contacted the Healthy Life Centre themselves could acknowledged their lifestyle problems, and thereby be readier to perform lifestyle change.

Our participants told about the importance of not relapsing from a changed habit, known in behaviour change theories staged that those who have performed lifestyles changes are working to prevent relapse [31]*.* Three months with intervention was said by participants to be to short time to make a lasting change, and the time aspect of incorporating changed behavior is important [31]. Help from health personnel is important to achieve long-lasting lifestyle change [32]. Participants in our study were also thankful that the Healthy Life Centre and accompanying personnel had been established to help. Another study found participants who had tried to change eating and/or activity habits several times felt unable to do so by themselves without any support, and that they wanted someone such as their primary health care providers to help them with the process of change [29].

### Emotional baggage

Studies have found that psychological and emotional overload from childhood can lead to negative coping behaviours and the suppression of negative emotions [33]. Furthermore, several studies have found associations between childhood mistreatment and unhealthy lifestyles as adults [34–38]. Childhood mistreatment can include neglect as well as physical, sexual, and emotional abuse [34, 37]. In our results, participants described different difficulties in childhood. Verbal abuse and lack of affection from parents were among the difficulties mentioned. In a person who lacks adequate emotional resilience, adverse childhood experiences can increase the risk of psychological and emotional distress with internal problems that may eventually cause a psycho-emotional overload [39]. Chronic stress over a long period, especially during vulnerable life stages, may have biological consequences in the long term [20]. Psychological distress from previous experiences in childhood can influence the ability to change negatively [20]. Previous experiences are key factors for self-efficacy [40]. Thus, experiences, which are negative, as they were for our participants, the self-efficacy, may be low. Consequently, the behavioural change gets harder to perform. A fundamental source of life satisfaction and emotional well-being is social support [41]. People with partners, family and friends who provide psychological and material support have better health than people with less social connections [42]. Studies have shown associations between social support and mental and physical health and variables for better health, such as physical activity, smoking and blood pressure [43]. Other studies found that those who failed to make lifestyle changes had more psychosocial crises such as grief, serious illnesses and personal or family difficulties [44].

### Changing ingrained habits

The complexity of the etiology in lifestyle issues may have been neglected in the past. We found psychological and emotional barriers for participants as important to change their habits when they were starting a lifestyle change program. Given the Healthy Life Centres’ salutogenic foundation, it is important to plan, act and intervene in accordance with the participants’ resistance resources [45]. Differences in resistance resources have implications for how participants handle health difficulties, diseases and negative life events [45]. Participants need to strengthen their resistance resources during the intervention period at Healthy Life Centres. Strong resistance resources are associated with high self-worth, inner strength, belief of being in control of change, social networks with a strong degree of affinity and good economic situations [45], which may not characterize this study’s participants. A successful change in lifestyle depends on the participants’ ability to use and rely on their resistance resources [45]. The outcome after an intervention period depends on whether Healthy Life Centres have strategies to enhance the resistance resources so that participants can change ingrained habits.

### Strengths and limitations

The strengths of the current study include the semi-structured interview form, where unexpected themes could arise. Participants themselves focused on their life stories and personal information to explain their situation, and thus, this became central to the study. In the study of complex human phenomena, qualitative interviews allow for ambiguity [15]. Lifestyle is an issue that affects so much of participants’ lives that it was important that the interviews allowed for such ambiguity.

The variation in age and gender in our study reflects the population that uses the Healthy Life Centres in the region. Then again, because Healthy Life Centres are a primary health care service, their challenge is embracing a large and differentiated user group with a broad range of participants in terms of lifestyle issues. If we had relied on a more selective sample, such as women aged 50–60 years, we might have had more focused answers, but the utility of our results in a clinical setting would be questionable.

## Conclusion

In Norway, Healthy Life Centres offer help in preventing and reducing lifestyle diseases. In our study, we found that participants who started a lifestyle intervention programme were stuck in old habits and had heavy emotional baggage. They wanted to change their old habits, but were unable to do so, even though they knew had information about healthy behaviours. Childhood and/or family, work and social life experiences were obstacles for lifestyle change. Previous negative experiences with accompanied psychological distress can influence the ability to change as self-efficacy may be low, which can make behavioural change hard to perform. Healthy Life Centres ought to consider the emotional burdens and previous life experiences of their clients as they work with participants to change old habits. However, our findings raise questions as to how health services should handle lifestyle change. Future studies should examine how lifestyle interventions in the Norwegian primary health care system are organized and how personnel can help participants with and promote lasting lifestyle changes. It remains to be seen who actually can benefit from Healthy Life Centres intervention programmes, and who will need other approaches to lifestyle changes, such as social or psychological interventions, or specialized health care treatment.
